# Survival status and predictors of mortality among road traffic accident adult patients admitted to intensive care units of referral hospitals in Tigray 2024

**DOI:** 10.1371/journal.pone.0308584

**Published:** 2025-03-03

**Authors:** Binyam Gebrehiwet Tesfay, Tensay Kahsay Welegebriel, Desta Hailu Aregawi, Mamush Gidey Abrha, Berhe Gebrehiwot Tewele, Fissha Brhane Mesele, Fiseha Abadi Gebreanenia, Kelali Goitom Weldu

**Affiliations:** 1 Adigrat University College of Medicine and Health Sciences, Adigrat, Ethiopia; 2 Mekelle University College of Health Sciences, Mekelle, Ethiopia; 3 Araya Kahsu Health Science College, Mekelle, Ethiopia; 4 Gembela Educational and Health Science College; 5 Axum University College of Health Science, Aksum, Ethiopia; University of Oulu: Oulun Yliopisto, FINLAND

## Abstract

**Background:**

Globally, road traffic accidents (RTAs) cause over 1.35 million deaths each year, with an additional 50 million people suffering disabilities. Ethiopia has the highest number of road traffic accidents, with over 14,000 people killed and over 45,000 injured annually. This study aimed to assess survival status and predictors of mortality among road traffic accident adult patients admitted to intensive care units of Referral Hospitals in Tigray, 2024.

**Methods:**

An institution-based retrospective follow-up study design was conducted from January 8, 2019, to December 11, 2023, on 333 patient charts. A bivariable Cox-regression analysis was performed to estimate crude hazard ratios (CHR). Subsequently, a multivariable Cox regression analysis was performed to estimate the Adjusted Hazard Ratios (AHR). Finally, AHR with p-value less than 0.05 was used to measure the association between dependent and independent variables.

**Result:**

The incidence of mortality for road traffic accident victims, was 21 per 1000 person-days observation with (95% CI: 16, 27.6) and the median survival time was 14 days. The predictors of mortality in this study were the value of oxygen saturation on admission ≤ 89% (AHR = 4.9; 95%CI: 1.4–17.2), Intracranial hemorrhage (AHR = 3.3; 95% CI: 1.02–11), chest injury (AHR = 3.2; 95%CI: 1.38–7.59), victims with age catgories of 31–45 years (AHR = 0.3; 95% CI: 0.1–0.88) and 46–60 years (AHR = 0.22; 95% CI: 0.06–0.89).

**Conclusion:**

A concerningly high mortality rate from car accidents were found in Referral Hospitals of Tigray. To improve the survival rates, healthcare providers should focus on victims with very low oxygen levels, head injuries, chest injuries, and older victims.

## Introduction

European Union define a road traffic accident as “An accident which occurred or originated on a way or street open to public traffic; resulted in one or more persons being killed or injured, and at least one moving vehicle was involved” [1].

Globally, according to the WHO 2018 report, Road traffic accidents (RTAs) represent more than 1.35 million deaths each year, accounting for 50 million incurring a disability; this makes it the 8th leading cause of death for all age groups [2].

Africa account for 20 percent of global road traffic deaths; the increased burden of road traffic injuries and deaths is attributed to countries’ income level. Furthermore, the average road traffic deaths in high-income countries account for 8.3 deaths per 100,000 people. On the contrary, the average road traffic deaths in low-income countries account for 27.5 deaths per 100,000, which indicates a more than three-fold increase [3,4]. In sub-Saharan Africa, 82% of road crash fatalities and injuries occur in the most economically productive age groups (15–64 years) [5].

In Ethiopia, RTA analysis from 2007/08 to 2017/18 revealed that the annual growth rate of road traffic accidents averaged around 9.16% per year [6]. The impact of RTA between 2016 and 2018 in Ethiopia shows that there were 14,194 fatalities, 22,647 serious injuries, and 21,159 minor injuries. Moreover, an average of 4,732 people die on the roads each year. Thus, this trend implies that more lives are lost due to road traffic injuries than other causes [7].

Furthermore, RTAs have increased excessively from time to time, which affects the most economically productive age group. Consequently, RTAs cause significant economic losses to individuals, their families, and to the development of the country in general [8,9].

A few studies revealed that factors such as head injury, thorax injury, abdominal injury, being a driver, the driver’s alcohol consumption, a severe Glasgow Coma Scale (GCS) score, systolic blood pressure, developed complications, and intracranial hemorrhage were associated with an increased risk of death among patients admitted to Hospitals with RTA [10–13].

Ethiopia has been striving to implement the African Union Road Safety Action Plan for 2011–2020, which focuses on post-crash management as well as other pertinent road traffic accident issues. Nevertheless, recent trends show no decrease in road traffic fatalities and injuries, highlighting the issue of inadequate facility-based trauma care [7]. Hence, the prevalence of road traffic deaths in Ethiopia remained at 31.5% [14].

The prolonged conflict and siege there have severely hampered healthcare infrastructure and trauma care systems, further amplifying the impact of RTAs. Despite the problem’s gravity, dependable statistical studies on RTAs, especially in Tigray, remain scarce. As far as I am aware, no published research exists in the region. This lack of data is concerning given the substantial public health burden posed by RTAs, both nationally and locally. This study aims to address this gap by assessing the survival status and identifying factors influencing mortality among adult RTA patients admitted to intensive care units (ICUs) in Tigray’s Referral Hospitals. Finally, this study will serve as a valuable baseline information for researchers in this critical area.

## Methods

### Study area

This study was conducted in Referral Hospitals within the Tigray Region of Ethiopia. Located in the country’s northern most corner. The region comprises two specialized hospitals served the region. Ayder referral hospital have three ICUs with 24 beds, serving to a population of 8 million across Tigray, Afar, and parts of the Amhara region. Aksum Comprehensive Specialized Referral Hospital, which established in 2016, and located 1010 kilometers from Addis Ababa, provided two ICUs with 16 beds, serving 3.5 million people from three Tigray zones. Moreover, clinical staff assigned in Ayder Comprehensive Specialized Referral Hospital Surgical ICU includes: anesthesiologist 6, X-ray technician 1, BSc Nurse 26, IPC nurse 1, clinical pharmacy 1, oxygen attendant 1, and 4 emergency and critical care specialists. In Aksum Comprehensive Specialized Referral Hospital Adult ICU, clinical staff included: anesthesiologist 2, BSc Nurse 16, emergency and critical care nurse 1, clinical pharmacy 1, and emergency and critical care specialist 1 [15].

### Study design and period

An institutional-based retrospective follow-up study design was conducted among RTA patients admitted to the ICU of the two comprehensive Referral Hospitals in Tigray between January 8, 2019, and December 11, 2023.

### Source population

All road traffic Accident patients admitted to intensive care units of Ayder and Aksum Comprehensive Specialized Referral Hospitals.

### Study population

All road traffic Accident patients from 08 January 2019–11 December 2023 admitted to adult intensive care units at Ayder and Aksum Comprehensive Specialized Referral Hospitals.

### Inclusion and exclusion criteria

#### Inclusion criteria

All medical records of road traffic Accident patients over 18 years old admitted to intensive care units of Ayder and Aksum Comprehensive Specialized Referral Hospitals from 08 January 2019–11 December 2023 were included.

#### Exclusion criteria

Incomplete patient chart.

### Sample size determination

The sample size was calculated using the STATA software version 17. The calculation was done based on the assumption that the type I error of 5%, power by 80% and a standard deviation of 0.5. The probability of event from a previous study conducted in Gondar University Hospital was 0.176 with hazard ratio of 2.26 [16]. The total sample size was 317 and considering 5% non-response rate the final sample size for this study were **333.**

### Sampling procedure and technique

Simple random sampling was used to select medical records from a sampling frame generated using MRNs in the record book. Data were accessed from both Referral Hospitals from January 5 to February 5, 2024. To select 333 medical records from the two Referral Hospitals, the full five-year records of RTA patients from January 8, 2019, to December 11, 2023, were isolated. Moreover, patient charts isolated from Ayder Comprehensive Specialized Hospital Surgical ICU and Aksum Comprehensive Specialized Hospital Adult ICU were 698 and 158 respectively. Finally, the sample was proportionally allocated based on the ratio of RTA patients in the two hospitals, with 272 for Ayder Comprehensive Specialized Hospital and 61 for Aksum Comprehensive Specialized Hospital. Computer-generated simple random sampling was used to select the study charts.

### Study variables

#### Dependent variable

Time to death

#### Independent variables

**Socio-demographic variables** include age, sex and residence address.

**Patients’ variables** include the availability of referral form, source of referral, duration to arrive this hospital, victim’s role, presence of co-morbidities and identified co-morbidity.

**Clinical related variables** include consciousness status at admission, oxygen saturation on admission, vital sign at admission, hemoglobin, the location of the injury, visceral organ injury, type of visceral organ injury, polytrauma, intracranial hemorrhage, presence of fracture, type of fracture, GCS, patient management, types of surgical procedure, intubation preformed, hospital acquired infection, disease acquired from hospital, reason for discharge from ICU and length of hospital stays.

### Operational definitions

**Time to death:** Time until an event occurs (i.e. Death) [17].

**Follow up time:** from the time of admission to the ICU due to road traffic accidents until either an event or censorship occurs.

**Event (1):** Death from road traffic Accident.

**Censored (0):** A subject without an event during the observation time was considered as censored if the patient is lost to follow up and transfer out to another service before developing the event or if the patient alive until the end of the study period that means up to 11 December 2023

**Time origin:** the time of admission to the ICU from road traffic accidents [17].

**Normal hemoglobin** based on WHO for adult men 13–17 g/dl, and for adult women 12-15g/dl.

**Anemia** was defined as having hemoglobin concentration <13 g/dL for adult men, and <12 g/dL for adult women [18]

### Data collection procedure and tools

Data were extracted from a patient record, a data extraction tool was adopted and modified based on similar studies referenced in the literature [8,11,16,19–26]. Patient records were initially observed, and an appropriate data extraction format was prepared in English. The tool included three sections: Socio-demographic variables, Patients’ variables and Clinical related variables. Data collectors used the tool to gather patient information from charts. Charts were retrieved using patient registration numbers from the electronic database system. One data clerk per hospital assisted with chart identification. Records (both baseline and follow-up) were reviewed before data collection. Subsequently, health professionals retrospectively reviewed all medical records of road traffic injury patients in the ICU who met inclusion criteria and were enrolled from January 8, 2019, to December 11, 2023. Data on patient deaths from road traffic accidents in the ICU was obtained from provider reports on medical cards.

### Data quality control

A pre-test of the data extraction format was conducted in Ayder referral hospital on a 5% random sample of the total charts before the actual study began. The collected data was checked for completeness, accuracy, and clarity. Each checklist item was assigned a code to facilitate tracing identified errors back to the corresponding records. Supervisors provided training to data collectors and closely monitored their work daily during data collection. Additionally, the principal investigator and supervisors reviewed the collected data daily.

### Data analysis

The data was checked for completeness and consistency and then entered into EPI-info version 7.2. It was subsequently exported to STATA version 17 for cleaning, coding, and analysis. During this process, the levels of missing values, the presence of influential outliers, multicollinearity, normality, and proportionality of hazards over time were all reviewed. To estimate survival time and cumulative probability of survival for patients with road traffic accident (RTA), descriptive statistics, Kaplan-Meier (KM) survival curves, life table and hazard functions were applied. Additionally, the Log rank test was used to compare survival curves for different categories of RTA patients. The Cox proportional hazards regression model assumptions were checked using the Schoenfeld residual test. Variables with P-values greater than 0.05 were considered to fulfill the assumption.

First, a bivariable Cox-regression analysis was conducted to estimate the crude hazard ratios (CHR). Each independent variable was tested against the dependent variable. Variables significant at P < 0.2 in the bivariate analysis, along with significant predictors identified in most relevant studies, were then included in the multivariable Cox regression model.

Subsequently, a multivariable Cox regression analysis was performed to estimate the Adjusted Hazard Ratios (AHR). A p-value less than 0.05 was considered as the threshold of statistical significance and AHR with a 95% Confidence Interval (CI) was used to measure the association between dependent and independent variables.

#### Ethical consideration

An official ethical clearance letter was obtained from the Mekelle University College of Health Sciences Institutional Review Board (IRB) on 14 December 2023 with Protocol of Approval MU-IRB 2130/2023. Moreover, the members of the IRB or ethics committee who approved this study were Professor Haftu Berhe Gebru, Dr Tesfay Gebregzabher Gebrehiwot, Dr Mengistu Welday Gebremihael, Dr Meskelu Kidu Weldetensae, Dr Abraha Hailu Weldegerima, Dr Gebretsadik Berhe, and Gidey Gebremeskel. Letters of cooperation were then written and addressed to both referral hospitals and relevant authorities. Upon receiving approval, communication was established with the medical directors, chief nursing directors, and intensive care unit heads at each referral hospitals.

Following these steps, the designated personnel proceeded with searching and obtaining the medical records of the selected samples. Throughout the process, greatest care was taken to ensure patient confidentiality. Given the retrospective nature of the study involving medical record review, the risk of harm to individual patients was minimal as long as confidentiality was strictly maintained. To guarantee confidentiality, all collected data were anonymized using codes and stored securely in a locked room before being entered into a password-protected computer system. Patient names were excluded from the data collection format entirely. The principal investigator remained the sole individual with access to the data. All methods were performed in accordance with declarations of Helsinki.

### Dissemination of results

A research report was submitted and presented to Mekelle University College of Health Sciences School of Nursing. Additionally, the study results were disseminated to the two Referral Hospitals in Tigray and the Tigray Regional Health Bureau.

## Result

### Socio demographic characteristics of the study subject

The study was conducted using the records of 333 road traffic accident victims with complete victim data charts. Among participants 272 (81.7%) were from Ayder Comprehensive Specialized Hospital. The study participants comprised 225 men (67.6%), resulting in a male-to-female ratio of 2.1:1. More than 80% of the participants were in the productive age group. The mean age was 35.52 ± 12.39 years (Table 1).

**Table 1:** Socio demographic characteristics of road traffic accident adult patients admitted to intensive care units of referral hospitals in Tigray 2024 (n = 333).

Variables	Category	Frequency	Percentage (%)
Age	18–30	146	43.8
	31–45	126	37.8
	46–60	47	14.1
	>60	14	4.2
Sex	Male	225	67.6
	Female	108	32.4
Residence	Urban	210	63.1
	Rural	123	36.9

### Patient`s related characteristics of the study subject

From the total 333 study subjects 68.8% arrived at both referral hospitals within 1 to 4 hours. According to recorded data, 73.6% of admitted RTA victims were referred from a healthcare facility, while 26.4% were directly transferred from the scene of injury. More than half (52.6%) of the study subjects were passengers. Out of 333 study subjects, 105 (31.6%) presented with comorbidity, and 66 (19.8%) presented with complications (Table 2).

**Table 2:** Patient’s related characteristics of road traffic accident adult patients admitted to intensive care units of referral hospitals in Tigray 2024 (n = 333).

Variables	Category	Frequency	Percentage (%)
Availability of referral form	Yes	245	73.6
	No	88	26.4
Source of referral	General Hospital	105	31.5
	PHCU	110	33
	Private	25	7.5
	Referral hospital	5	1.5
Hospital arrival Duration	≤ 1 hr.	49	14.7
	1–4 hr.	229	68.8
	4–24 hr.	44	13.2
	≥ 24 hr.	11	3.3
Victim role	Driver	50	15
	Passenger	175	52.6
	Pedestrian	108	32.4
Presence of comorbidity	Yes	171	51.4
	No	162	48.6
Identified comorbidity	ARDS	21	6.3
	Asthma	22	6.6
	CHF	22	6.6
	DM	11	3.3
	HIV	11	3.3
	HTN	18	5.4
**Identified complications**	Fat embolism	25	7.5
	Hypovolemic Shock	41	12.3

Key PHCU; Primary Health Care Unit.

### Clinical characteristics of the study subject

Out of 333 subjects head injury comprising 276 (82.9%), polytrauma accounted for 132 (39.6%), and chest injury also comprising 152 (45.6%). Based on pulse oximetry, the oxygen saturation of patients on admission with less than or equal to 89% were 74 patients (22.2%) and greater than 89% were 259 patients (77.8%). From the total 238 patients had normal blood pressure. Intracranial hemorrhage was present in 75 patients (22.5%). Moreover, out of the observed 333 road traffic accidents, fracture accounted for 326 (97.9%). From the total 326 fracture extremity bone fracture was 173 (52%). Finall, the most frequently performed surgical procedure was thoracostomy, 125 (37.5%) (Table 3).

**Table 3:** Clinical characteristics of road traffic accident adult patients admitted to intensive care units of referral hospitals in Tigray 2024 (N = 333).

Variables	Category	Frequency	Percentage (%)
Glasgow Coma Score	GCS <8: Severe Head Injury	333	100
Oxygen saturation on admission	≤ 89	74	22.2
	˃ 89	259	77.8
Blood pressure of a patient at admission	≤ 89/59	77	23.1
	90/60–139/89	238	71.5
	≥ 140/90	18	5.4
Respiratory rate of a patient at admission	˃ 24	168	50.5
	16–24	165	49.5
Pulse rate of a patient at admission	˃ 99	164	49.2
	60–99	169	50.8
Hemoglobin	Normal value	218	65.5
	Abnormal value	115	34.5
Extremity injury	Yes	173	52
	No	160	48
Head injury	Yes	276	82.9
	No	57	17.1
Spinal cord injury	Yes	52	15.6
	No	281	84.4
Abdominal injury	Yes	36	10.8
	No	297	89.2
Chest injury	Yes	152	45.6
	No	181	54.4
Visceral Organ injury	Yes	168	50.5
	No	165	49.5
Types of visceral organ injury	Heart	12	3.6
	Lung	115	34.5
	Liver	19	5.7
	Spleen	22	6.6
Polytrauma	Yes	132	39.6
	No	201	60.4
Intracranial hemorrhage	Yes	68	20.4
	No	265	79.6
Presence of bone fracture based on X-ray	Yes	326	97.9
	No	7	2.1
Extremity fracture	Yes	173	52
	No	160	48
Ribs fracture	Yes	128	38.4
	No	205	61.6
Spinal fracture	Yes	42	12.6
	No	291	87.4
Simple linear skull fracture	Yes	144	43.2
	No	189	56.8
Basal Skull fracture	Yes	57	17.1
	No	276	82.9
Patient`s management	Surgical & Conservative management	321	96.4
	Conservative management only	12	3.6
Types of surgical procedure performed for Extremity fracture	Internal & External Fixation	80	46.2
	POP	47	27
	Wiring & pin traction	46	26.8
Debridement performed	Yes	239	71.8
	No	94	28.2
Craniotomy performed	Yes	35	10.5
	No	298	89.5
Laparotomy performed	Yes	33	9.9
	No	300	90.1
Thoracostomy performed	Yes	125	37.5
	No	208	62.5
Intubation preformed	Yes	333	100
Hospital acquired infection	Yes	246	73.9
	No	87	26.1
Disease acquired from hospital	VAP	29	11.8
	Sepsis	203	82.5
	Bed sore	14	5.7
Discharge status from ICU	Died (1)	53	15.9
	Censored (0)	280	84.1

### Incidence of mortality and survival status

A total of 333 patients were followed for a minimum of 1 day and a maximum of 14 days, and the median survival time was 14 days. During the follow-up period, 53 (15.9%) died and 280 (84.1%) were censored with 258(92.1%) transfer into surgical ward and 22(7.9%) were referred. The total time at risk for the 333 patients was 2516 person-days, with an overall incidence of mortality among admitted RTA victims of 21 per 1000 person-days of observation (95% CI: 16, 27.6 per 1000 person-days of observation).

From the total 53 died, 14 (26.42%) were female, and 39 (73.58%) were male. The incidence of mortality for the male population was higher (23/1000 person-days; 95% CI: 16, 30/1000 person-day observation) than females.

Drivers had a higher mortality rate than passengers and pedestrians (31/1000 person-days; 95% CI: 16, 57/1000 person-days observation). Among victims, the incidence of mortality was highest (135/1000 person-days; 95% CI: 97, 188/1000 person-days observation) for those arriving at the healthcare facility late (between 4 and 24 hours after the accident).

Victims with polytrauma had the highest incidence of mortality (34.7/1000 person-days; 95% CI: 25, 48/1000 person-days observation). Another variable significantly associated with death among road traffic accident victims was extremity fracture. Interestingly, victims without extremity fracture also had a high incidence of mortality (31/1000 person-days; 95% CI: 22, 43/1000 person-days observation).

**Fig 1:**
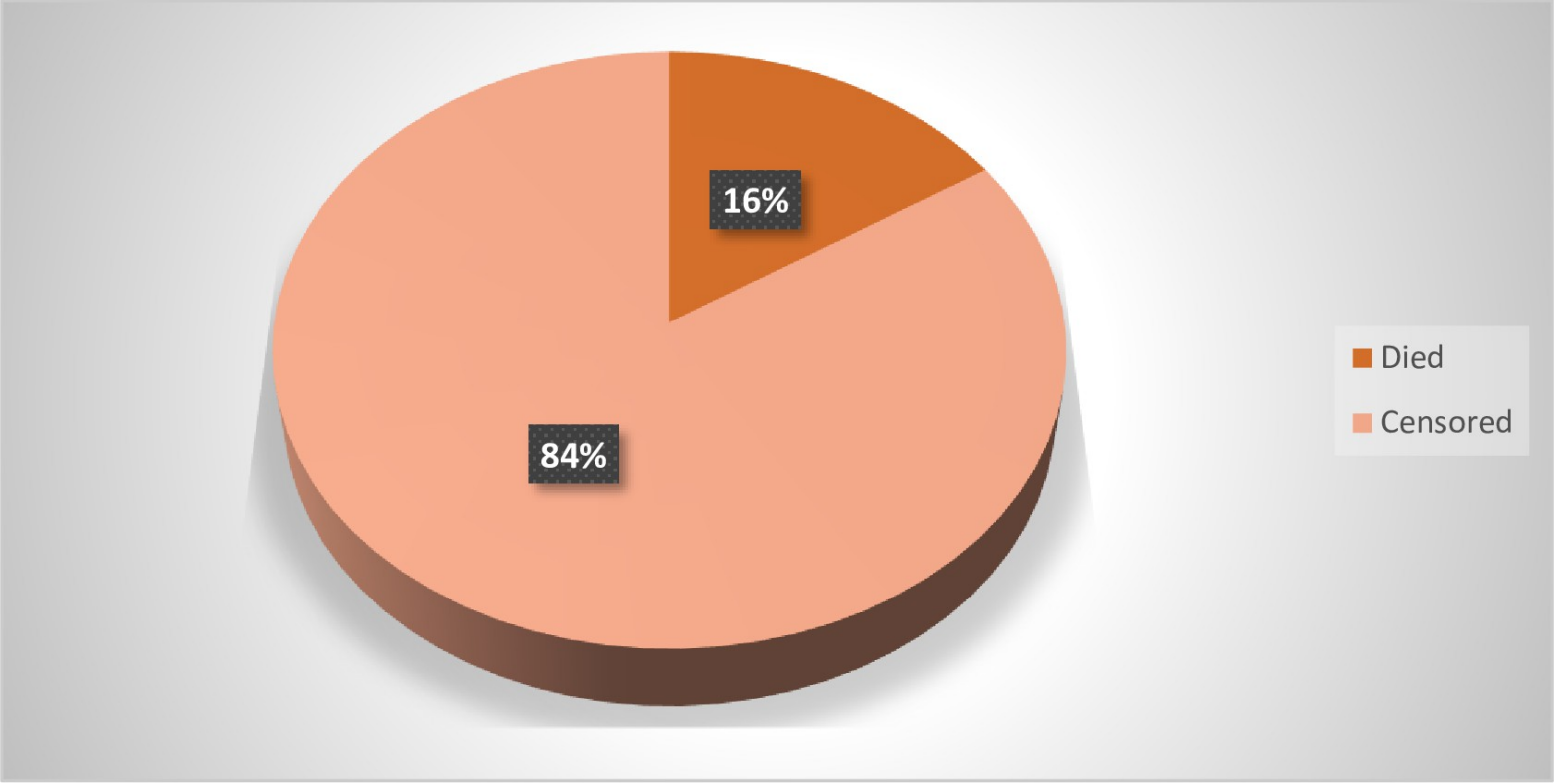
Proportion of road traffic accident adult patients admitted to intensive care units of referral hospitals in Tigray 08 January 2019–11 December 2023.

Among those with a history of low blood pressure (less than 89/59 mmHg), the incidence of mortality was high (74/1000 person-days; 95% CI: 55, 100/1000 person-days observation). Ventilation-associated pneumonia, a hospital-acquired disease, also had a high incidence of mortality (43/1000 person-days; 95% CI: 23, 80/1000 person-days observation) (Fig 1).

The median survival time of road traffic accident adult patients admitted to ICU of referral hospital in Tigray was 14 days (Table 4).

**Table 4:** Overall life tables of road traffic accident adult patients admitted to intensive care units of referral hospitals in Tigray 08 January 2019–11 December 2023 (n = 333).

Interval	Beg. Total	Death	Lost	Survival	Std. Error	[95% Conf. Int.]
1–2	333	4	0	0.9880	0.0060	0.9683–0.9955
2–3	329	6	0	0.9700	0.0094	0.9449–0.9837
3–4	323	2	21	0.9638	0.0103	0.9371–0.9793
4–5	300	11	22	0.9271	0.0147	0.8922–0.9510
5–6	267	7	34	0.9011	0.0172	0.8614–0.9299
6–7	226	8	34	0.8666	0.0204	0.8206–0.9015
7–8	184	5	38	0.8404	0.0229	0.7893–0.8800
8–9	141	2	22	0.8274	0.0243	0.7735–0.8696
9–10	117	0	17	0.8274	0.0243	0.7735–0.8696
10–11	100	0	20	0.8274	0.0243	0.7735–0.8696
11–12	80	2	18	0.8041	0.0287	0.7405–0.8537
12–13	60	0	14	0.8041	0.0287	0.7405–0.8537
13–14	46	2	24	0.7568	0.0422	0.6620–0.8285
14–15	20	4	16	0.5046	0.1068	0.2853–0.6886

The cumulative proportion of surviving at 1 day of admission or injury was 98% (95% CI: 96.83%, 99.55%) and similarly, it was, 90% (95% CI: 86.14%, 92.99%), 82% (95% CI: 77.35%, 86.96%) and 50% (95% CI: 28.53%, 68.86%) at the end of 5^th^, 10^th^, and 14^th^ respectively.

**Fig 2:**
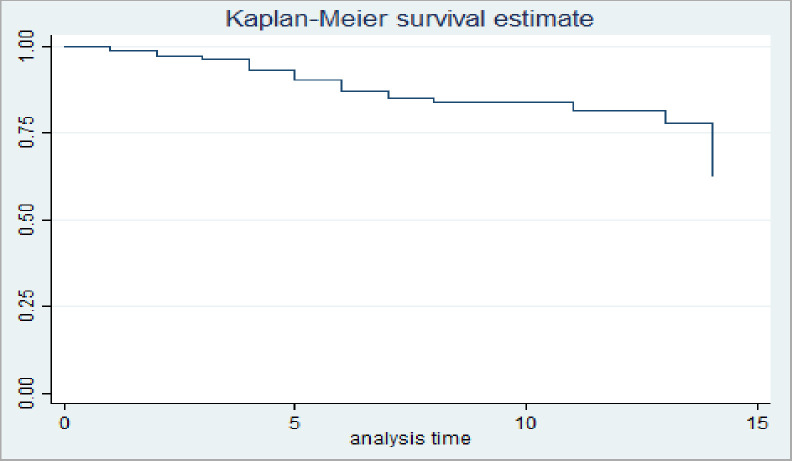
Overall Kaplan Meier estimation their survival among road traffic accident adult patients admitted to intensive care units of referral hospitals in Tigray 08 January 2019–11 December 2023.

At first day of follow-up, the probability of survival for those RTA victims with oxygen saturation ≤89% and >89% was 95.95% and 99.61%, respectively. Similarly, the on the first day of survival probability for RTA victims with intracranial hemorrhage was 95.59%, and 99.62% without intracranial hemorrhage. Finally, the 1st day of survival probability for RTA victims with chest injury was 97.37%, and the 1st day of survival probability for RTA victims without chest injury was 100% (Fig 2).

**Fig 3:**
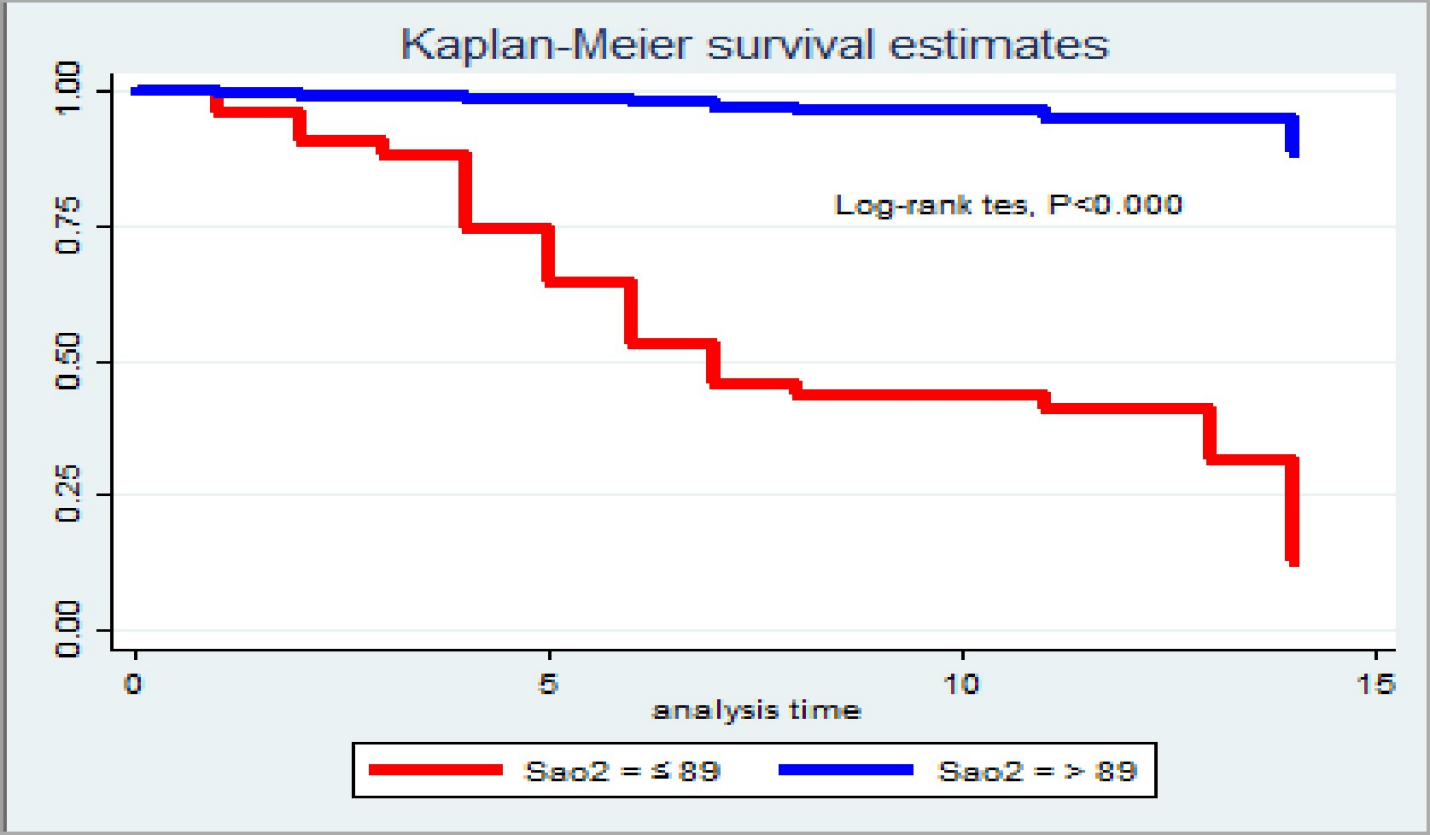
Kaplan Meier survival estimate for categorized Oxygen saturation among road traffic accident adult patients admitted to referral hospitals in Tigray 08 January 2019–11 December 2023.

The incidence of mortality among road traffic accident victims with oxygen saturation on admission ≤89% was significantly different and higher than those of oxygen saturation on admission >89; similarly it was also true in case of Intracranial hemorrhage, age of victims and Chest injury (Fig 3).

There was statistically significant difference in death of accident victims with oxygen saturation on admission >89 as compared to those with ≤ 89. There is also statistically significant difference in mortality among the two categories of intracranial hemorrhage, and chest injury.

Kaplan-Meier survival curve with together with the log-rank test was fitted to test for the occurrence of death among categorical explanatory variables. There was high incidence of mortality among victims with decreased Oxygen saturation (log-rank test, χ2 135.5 and chest injury (log-rank test, χ2 20.7) with P-value of <0.000 (Table 5).

**Table 5:** Comparison of mortality among categories of predictor variables by using log-rank test of road traffic accident adult patients admitted to referral hospitals in Tigray 08 January 2019–11 December 2023 (n = 333).

Variables	Category	IMR/100 pdo	Log-rank(χ2)	p-value
Residence	Urban	1.7 2.6	2.82	0.093
	Rural			
Age	18–30 years	2.1 1.4 2.2 10	27.6	<0.000
	31–45 years			
	45–60 years			
	>60 years			
Hospital arrival Duration	≤ 1 hr.	2.1 0.32 13.5 7.1	192.66	<0.000
	1–4 hr.			
	4–24 hr.			
	≥ 24 hr.			
Victim role	Driver	3.1 1.26 3.04	9.77	<0.0075
	Passenger			
	Pedestrian			
Oxygen saturation on admission	≤ 89	8.8 0.4	135.5	<0.000
	˃ 89			
Blood pressure on admission	≤ 89/59	7.4 0.2 6.4	113.74	<0.000
	90/60–139/89			
	≥ 140/90			
Intracranial hemorrhage	No	0.3 10.5	184.2	<0.000
	Yes			
Hemoglobin on admission	Normal	0.3 5.9	79.87	<0.000
	Abnormal			
Chest injury	No	0.9 3.5	20.7	<0.000
	Yes			
Polytrauma	No	1.14 3.47	15.43	<0.0001
	Yes			
Basal skull fracture	No	0.7 9.7	120.43	<0.000
	Yes			

### Predictors of mortality

Factors with a p-value less than 0.2 in the bivariable Cox proportional hazard model were included in the multivariable analysis. Finally, the predictors of mortality in the final model were the value of oxygen saturation on admission ≤ 89 (AHR = 4.9; 95%CI: 1.4–17.2), Intracranial hemorrhage (AHR = 3.3; 95% CI: 1.02–11), chest injury (AHR = 3.2; 95%CI: 1.38–7.59), victims with age of 31–45 years (AHR = 0.3; 95% CI: 0.1–0.88) and 46–60 years (AHR = 0.22; 95% CI: 0.06–0.89) (Table 6).

**Table 6:** Bivariable cox regression analysis for independent predictors of time to death among road traffic accident adult patients admitted to referral hospitals in Tigray 08 January 2019–11 December 2023 (n = 333).

Variables	Categories	Death	Censored	CHR (95% CI)	P-value
Age	18–30	23	123	0.18 (0.08–0.42)*	P<0.001
	31–45	15	111	0.12 (0.05–0.32)*	P<0.001
	46–60	8	39	0.19 (0.69–0.54)*	P<0.002
	>60	7	7	1	
Residence	Urban	28	182	0.63 (0.37–1.1)*	P<0.101
	Rural	25	98	1	
Duration	≤1hr	7	42	1	
	1–4	6	223	0.14 (0.05–0.4)*	P<0.001
	4–24	35	9	6.14 (2.7–13.8)*	P<0.000
	>24	5	6	3.46 (1.1–10.9)*	P<0.034
BP	≤ 89/59	40	37	1	
	90/60–139/89	5	233	0.037(0.02–0.9)*	P<0.000
	≥ 140/90	8	10	0.9 (0.42–1.93)	P<0.799
Oxygen saturation on admission	≤ 89	44	30	19.6 (9.6–40.4)*	P<0.000
	˃ 89	9	250	1	
Intracranial hemorrhage	Yes	46	22	32(14.4–71.07)*	P<0.001
	No	7	258	1	
Hemoglobin at admission	Abnormal	47	68	16.5(7.07–38.8)*	P<0.000
	Normal	6	212	1	
Chest injury	Yes	40	112	3.7 (2.02–7.06)*	P<0.000
	No	13	168	1	
Polytrauma	Yes	36	96	3 (1.66–5.3)*	P<0.000
	No	17	184	1	
Extremity fracture	Yes	19	154	0.43(0.24–0.75)*	P<0.003
	No	34	126	1	
Basal Skull fracture	No	17	259	0.08(0.04–0.15)*	P<0.001
	Yes	36	21	1	
Linear skull fracture	Yes	17	127	0.58(0.32–1.04)*	P<0.069
	No	36	153	1	
Craniotomy	No	26	272	0.07(0.41–0.13)*	P<0.001
	Yes	27	8	1	
Debridement	Yes	28	211	0.38(0.22–0.66)*	P<0.001
	No	25	69	1	

Variables with p-value less than 0.2 in bivariable analysis were taken to multivariable cox regression analysis.

**Fig 4:**
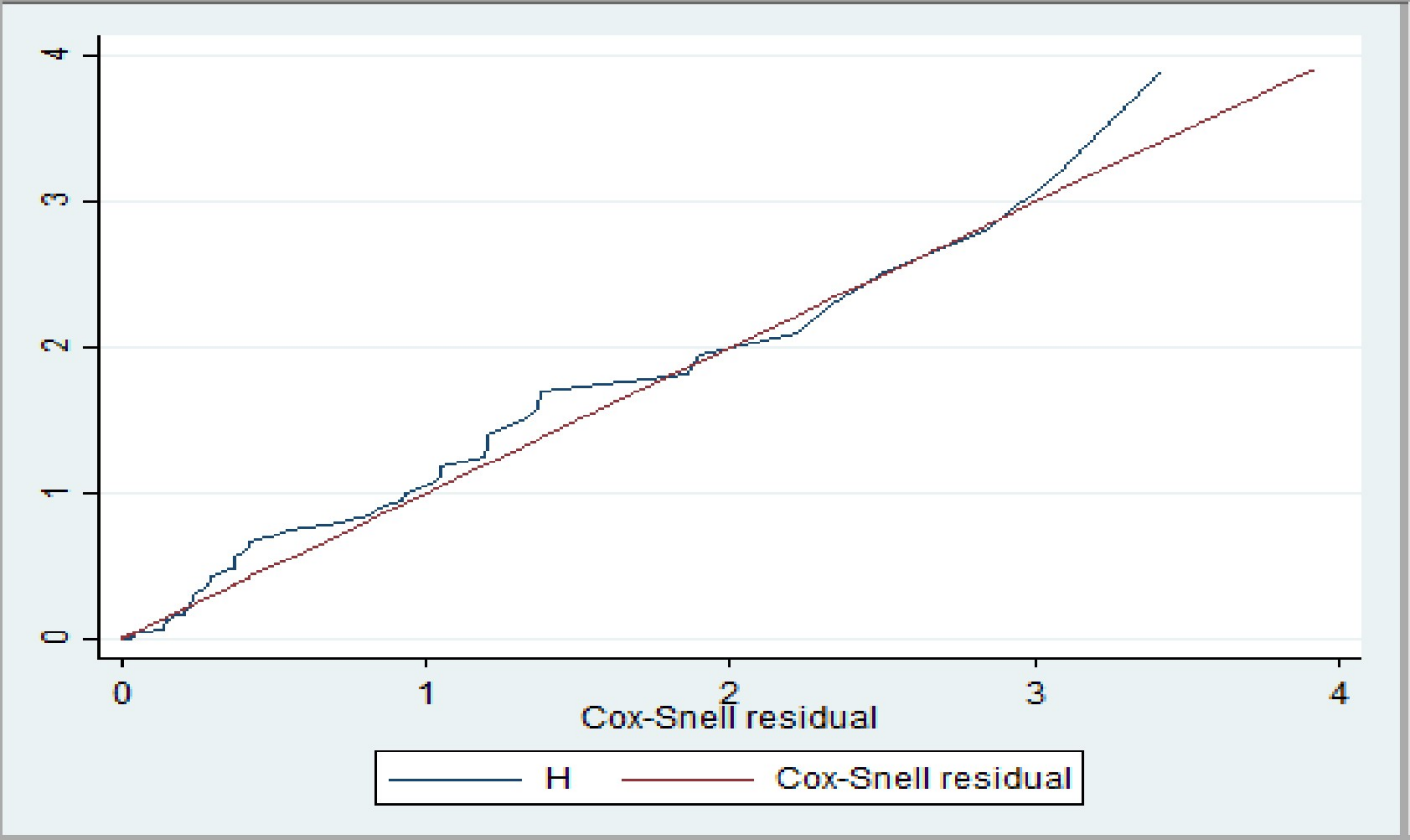
Nelson-Aalen cumulative hazard graph against Cox-Snell residual on road traffic accident adult patients admitted to referral hospital in Tigray 08 January 2019–11 December 2023 (n = 333).

The cox-proportional hazard assumption was checked by using overall schoenfeld global test for full model and it was met (p = 0.8014). All covariates are met for proportional hazard assumption. Residuals were checked using the goodness-of-fit test by Cox-Snell residuals. The final model fits the data as in the figure below by which the hazard function follows 45° (Fig 4).

Finally, the predictors of mortality in the multivariable Cox regression analysis were RTA victims with an oxygen saturation at admission of ≤89%. The hazard of death increased by five times (adjusted hazard ratio [AHR] = 4.9; 95% CI: 1.4–17.2) compared to victims with oxygen saturation greater than 89%. Patients with intracranial hemorrhage had a three times higher risk of death (AHR = 3.3; 95% CI: 1.02–11) compared to those without intracranial hemorrhage. Victims aged 31–45 and 46–60 had a decreased hazard of death by 70% (AHR = 0.3; 95% CI: 0.1–0.88) and 78% (AHR = 0.22; 95% CI: 0.06–0.89), respectively, compared to those over 60 years old. Finally, RTA victims with chest injury had a three times increased hazard of death (AHR = 3.2; 95% CI: 1.38–7.59) compared to victims without chest injury (Table 7).

**Table 7:** Multivariable cox regression analysis for independent predictors of time to death among road traffic accident adult patients admitted to referral hospital in Tigray 08 January 2019–11 December 2023 (n = 333).

Variables	Category	Death	Censored	AHR (95% CI)	P-value
Oxygen saturation on admission	≤ 89	44	30	4.9(1.4–17.2)	P<0.013
	˃ 89	9	250	1	
Intracranial hemorrhage	Yes	46	22	3.3(1.02–11)	P<0.047
	No	7	258	1	
Chest injury	Yes	40	112	3.2(1.38–7.59)	P<0.007
	No	13	168	1	
Age of victim	18–30	23	123		
	31–45	15	111	0.30(0.1–0.88)*	P<0.029
	46–60	8	39	0.22(0.06–0.9)*	P<0.033
	>60 years	7	7	1	

## Discussion

This study found that the death rate from RTA was 21 per 1000 person-days of observation. This rate is higher than previous study conducted in Ethiopia, which reported rates of 2.9 per 10,000 person-hours of observation [16]. This is likely due to our study only focused on critical patients and the previous study was done in the Emergency department with follow up time of 24 hours. Additionally, the Tigray conflict severely affected the trauma care system, including its ability to treat RTA victims admitted to the ICU.

The median survival time RTA victims was 14 days which is shorter than the study conducted in Congo 18 days [13]. This might be because our study only looked at patients admitted to the ICU, while the D.R. Congo study included all road traffic patients admitted to hospitals during the study period. Furthermore, the follow up period of study conducted D.R. Congo were extended to six years with different area of data collection or setting besides the ICU.

The incidence of mortality among the male population (23/1000 person-days observation) was higher than among the female population (17/1000 person-days observation) in our study. This result is similar to a study conducted in Ethiopia, where the mortality rate among males was 8.0/10,000 and among females was 4.5/10,000. This is likely because the male population engages in more outdoor activities than the female population, including travel by automobile over long distance [12].

The current study revealed that the incidence of mortality among RTA victims higher among age group greater than 60 years (100/1000 person-days observation) which was inline with studies conducted in Sidama [12]. The possible reason behind this fact is the older age victms are vulerable for chronic diseases and hospital acquired infections due to deterioration of immunity system and normal physiology of the body this reason increase the incidence of mortality among older age.

According to this study, the incidence of mortality was high (135/1000 person-day observation) among patients who arrived late (4–24 hours) to the health facility. This result consistent with study done in Ethiopia, where over 50% of deaths among late-arriving victims comprised a total of 26 (6%) deaths [27]. The possible explanation might be many victims lost valuable time at other institutions, especially primary health care settings, without getting adequate treatment before arriving at referral hospitals. This explanation was supported by the ’golden hour’ concept for managing emergency and critical victims [4].

The finding of this study confirmed that low oxygen saturation increases the risk of death compared to normal oxygen saturation with a hazard ratio of 4.9. This finding was consistent with a study conducted in China where patients with low oxygen saturation were found to have a higher risk of death with a hazard ratio of 2.2 [28]. A possible explanation for low oxygen saturation is that severe bleeding as a result of RTA can lead to a lack of red blood cells, which are vital for carrying oxygen to the body’s tissues. This deficiency can result in insufficient oxygen reaching vital organs leading to death. This explanation was supported by Medical Surgical Nursing Book [29].

Victims aged 31–45 and 46–60 had decreased hazards of death by 70% (AHR = 0.3; 95% CI: 0.1–0.88) and 78% (AHR = 0.22; 95% CI: 0.06–0.89), respectively, compared with those aged over 60 years. A possible explanation for the increased risk of death in older adults is changes in bone density and muscle strength. Additionally, age-related changes in physiology or pre-existing medical conditions can further increase their risk.

This study indicates that intracranial hemorrhage significantly increases the hazard of death among RTA victims compared to those without it with harard ratio of 3.3. This finding was inline with a study conducted in Taiwan with harard ratio of 3.1 [10]. The likely explanation is that intracranial hemorrhage causes blood to pool between the brain and the skull, which can prevent oxygen from reaching the brain. This life-threatening condition increases the hazard of death among road traffic accident victims This explanation was supported by Medical Surgical Nursing Book [29].

This study identified that from admitted RTA victims, 40 (12%) die as a result of chest injury with a hazard ratio of 3.2. This means that the risk of death was higher for those with chest injuries compared to their counterparts. This finding was higher than, studies conducted in Saudi Arabia (4.8%), and Egypt (6.9%) [30–32]. Medical Surgical Nursing Book clarified that the thoracic cavity houses vital organs like the lungs and heart. Injuries to this area can lead to life-threatening complications such as airway obstruction, massive hemothorax, pneumothorax, cardiac dysfunction, and damage to large intrathoracic vessels. Death is imminent if these conditions occur [29].

### Limitation of the study

#### Data collection bias

Even with well-defined data extraction tools, bias can arise during data collection. Identifying missing data or interpreting ambiguous entries may introduce inconsistencies, even with training and supervision.

#### Exclusion of post-discharge deaths

Our study excluded deaths occurring after discharge from the ICU. This exclusion might underestimate the overall mortality rate and its association with various factors.

## Conclusion

This study found a high number of deaths among RTA victims admitted to Referral Hospitals in Tigray with over all median survival time of 14 days. This study also identified several factors that increased the risk of death, including being older than 60 years old, having low oxygen levels, chest injuries, and intarcaranial hemorrage. Additionally, the study observed that people who arrived at the hospital later after the accident and men were more likely to be affected by RTAs.

Finall, researchers who are intersted in the sudy area; it is better to come with prospective designs, broader inclusion criteria, and comprehensive data collection.

## Supporting information

S1 Dataset (XLSX)

S1 Annex (DOCX)

## Abbreviations

AHR: Adjusted Hazard Ratio
CHR: Crude Hazard Ratios
GCS: Glasgow Coma Score
ICU: Intensive Care Unit
IMR: Incidence Mortality Rate
Pdo: Person Days Observation
Pho: Person Hours Observation
RTA: Road Traffic Accident
RTI: Road Traffic Injury
TBI: Traumatic Brain Injury
VAP: Ventilation Associated Pneumonia
