# Supplementary material for: Survival status and predictors of mortality among road traffic accident adult patients admitted to intensive care units of referral hospitals in Tigray 2024
Source: PLoS One. 2025 Mar 3;20(3):e0308584. doi: 10.1371/journal.pone.0308584 (PMC11875276; doi:10.1371/journal.pone.0308584)
Supplement: S1 Annex — (DOCX) [file pone.0308584.s002.docx]

**ANNEX : Data Extraction Format (Checklist)**

This tool is prepared for the collection of socio-demographic, patient’s and clinical related information that are important for the assessment of survival status and predictors of mortality among road traffic accident adult patients admitted to intensive care units of referral hospital in Tigray, Ethiopia 2024. All this information were retrieved from the Patients medical records without mentioning the name of clients.

**Part I: Socio demographic characteristics**

Code No._____________ MRN_____________ Hospital name___________________________

| SNO | Socio demographic variables | Response |  |
| --- | --- | --- | --- |
| 101 | Age | ________In years |  |
| 102 | Sex | 1, Male 2. Female |  |
| 103 | Residency | 1. Urban 2. Rural |  |

**Part II: Patient`s related variables**

| SNO | **Variables** | Response |  |
| --- | --- | --- | --- |
| 201 | Availability of referral form | 1. Yes 2. No | If yes fill the next response |
| 202 | Source of referral | 1. Referral from general hospitals 2. Referral from private hospitals 3. Referral from PHCU 4. Referral from Referral Hospital |  |
| 203 | Duration to arrive this  Hospital? | ____________ (in hour) |  |
| 204 | Victim’s role | 1. Passenger 2. Pedestrian 3. Driver 4. Other_______ | If others specify_____ |
| 205 | Presence of Co-morbidities | 1. Yes 2. No | If yes go to Q 206 |
| 206 | Co-morbidities identified | 1. Diabetes mellitus  2. Hypertension  3. HIV/AIDS  4. CHF 5. Others____ | If others specify_____ |
| 207 | Identified complication | 1. Hypovolemic shock 2. Fat embolism 3. Other_______ |  |
| 208 | Admission date: | (dd/mm/yy) |  |

**Part III: Clinical related variables**

| SNO | **Variables** | Response | Remark |
| --- | --- | --- | --- |
| 301 | Consciousness status at admission | 1. Conscious 2. unconscious |  |
| 302 | Oxygen saturation on admission | ­­___________% |  |
| 303 | Vital signs of a patient at admission | 1. BP________ 2. RR_________ 3. PR__________ 4. T 0__________ |  |
| 304 | Hemoglobin | ____________g/dl |  |
| 305 | Location of injury | 1. Extremities 2. Head 3. Abdomen 4. Chest 5. Spinal cord 6. Other | If others specify_____ |
| 306 | Polytrauma | 1. Yes 2. No |  |
| 307 | Visceral Organ injury | 1. Yes 2 No |  |
| 308 | Type of visceral Organ injury | 1. Spleen 2. Liver 3. Heart 4. Lung 5. Other |  |
| 309 | Intracranial hemorrhage | 1. Yes 2. No |  |
| 310 | Presence of bone fracture based on X-ray | 1. Yes 2. No | If yes fill the next response |
| 311 | Particular bone fractured? | 1. Extremity 2. Ribs 3. Skull 4. Spinal 5. Multiple 6. Other ___ | If others specify_____ |
| 312 | Patient`s management | 1. Surgical and Conservative management 2. Conservative management only (fluid and antibiotic administration) 3. Others (specify)___________ | If the repose is Surgical fill the next response |
| 313 | Types of surgical procedure performed | 1. Debridement 2. Craniotomy 3. POP 4. Internal & External Fixation 5. Wiring & pin traction 6. Thoracostomy 7. Laparotomy 7. Others | If others specify_____ |
| 314 | Intubation preformed | 1. Yes 2. No |  |
| 315 | Glasgow Coma Score | 1. GCS 14 -15: Mild Head Injury 2. GCS 9-13: Moderate Head Injury 3. GCS < 8: Severe Head Injury |  |
| 316 | Hospital acquired infection | 1. Yes 2. NO | If yes go to Q317 |
| 317 | Disease acquired from hospital | 1. Ventilation associated Pneumonia  2. Bed sore  3. Sepsis 4. Others__________ | If others specify_____ |
| 318 | Date of discharge | (in dd/mm/yy) |  |
| 319 | Discharge status from ICU | 1. Died (1) 2. Censored (0) | If censored go to Q320 |
| 320 | Types of censorship | 1. Transferred out 2. Transferred in 3. Referred |  |
| 321 | Length of stay in ICU | __________Days |  |

Name of data collector _________________sign ___________date _____________

Name of supervisor ____________________sign ___________date _____________
